# Aqueous humor cytokine levels in patients with diabetic macular edema refractory to anti-VEGF treatment

**DOI:** 10.1371/journal.pone.0203408

**Published:** 2018-09-11

**Authors:** Jin-woo Kwon, Donghyun Jee

**Affiliations:** Department of Ophthalmology and Visual Science, St. Vincent’s Hospital, College of Medicine, Catholic University of Korea, Suwon, Kyunggi-do, Korea; University of Florida, UNITED STATES

## Abstract

**Purpose:**

To determine aqueous cytokines association with response to intravitreal bevacizumab (IVB) injection in diabetic macular edema (DME).

**Method:**

We compared the concentrations of IL (interleukin)-1β, IL-2, IL-8, IL-10, IL-17, placental growth factor (PlGF), and vascular endothelial growth factor (VEGF) in the aqueous humor of 64 naïve DME patients with those of 13 cataract patients. Factors associated with central subfield thickness (CST) in DME patients were identified. DME patients were then subgrouped in terms of responsiveness to three IVB injections; cytokine concentrations were compared, and factors associated with responsiveness were identified.

**Results:**

Levels of IL-2, IL-8, PlGF, and VEGF were significantly elevated in DME patients (p = 0.007, p < 0.001, p < 0.001, and p = 0.004 respectively). Regression analysis showed that the preoperative CST was associated with the preoperative best-corrected visual acuity and the aqueous IL-10 level (p < 0.001, p = 0.006, respectively). Of the 64 DME patients, 28 (43.75%) exhibited either CST < 300 μm or reduction in CST ≥ 50 μm after three consecutive IVB injections. On sub-group analysis, the mean IL-8 concentration was higher in the refractory group than in the responsive group, and multivariate logistic regression analysis showed that the IL-8 was the only factor associated with responsiveness (OR = 0.95, p = 0.017).

**Conclusions:**

The IL-8 concentration in the aqueous humor was associated with responsiveness to IVB in DME patients.

## Introduction

Diabetic retinopathy (DR) is one of the most significant causes of visual impairment worldwide, despite advances in laser and surgical treatments.[1–5] The visual impairment associated with DR is principally attributable to neovascularization or diabetic macular edema (DME).[5–7] The early pathogenesis of DME features damage to the blood—retina barrier, characterized by loss of pericytes and endothelial tight junctions induced by metabolic alterations and inflammation.[7, 8] DME is affected by not only inflammatory and glial cells but also the expression levels of inflammatory molecules, including interleukins (ILs), vascular endothelial growth factor (VEGF), tumor necrosis factor-α, transforming growth factor-β, and matrix metalloproteinases (MMPs).[8–10] Based on fundamental studies of the effects of VEGF and anti-VEGF antibodies, several types of anti-VEGF agents have been shown to effectively treat DME.[11, 12] Recently, intravitreal steroid implants have also been proven to reduce central subfield thickness (CST) and improve visual acuity in DME patients.[13, 14]

Many studies have shown that the aqueous humor of DME patients contains elevated levels of inflammatory cytokines, growth factors, and MMPs, associated with retinal status.[15–17] The individual roles played by these factors in pathogenesis remain unclear; many studies have sought to determine the detailed mechanisms of action. We thus compared the levels of IL-1β, IL-2, IL-8, IL-10, IL-17, placental growth factor (PlGF), and VEGF in the aqueous humor of 64 naïve DME patients with those of a control group. We also identified factors associated with responsiveness to intravitreal bevacizumab (IVB).

## Methods

We compared IL-1β, IL-2, IL-8, IL-10, IL-17, PlGF, and VEGF levels in the aqueous humor of naïve DME patients with type II diabetes mellitus (DM). We followed all relevant tenets of the Declaration of Helsinki. The protocol was approved by the Institutional Review/Ethics Board of the Catholic University of Korea. All methods were performed in accordance with the relevant guidelines and regulations by the protocol. All participants gave written informed consent to the use of their clinical records.

We enrolled 64 naïve DME eyes (of 64 patients) of CST > 300 μm; 13 diabetes-free patients with cataracts served as controls. The exclusion criteria included glaucoma, retinal degeneration, and macular edema attributable to other causes including an epiretinal membrane or vitreo-macular traction. We excluded eyes with concurrent diseases such as retinal vascular occlusion, and eyes with histories of prior ocular conditions, uveitis, or intraocular surgery that could influence enzyme levels in the aqueous humor.

Every patient underwent a full ophthalmological examination including measurement of visual acuity, refraction, and intraocular pressure (IOP), in addition to a dilated fundus examination. All eyes were classified using the Early Treatment of Diabetic Retinopathy criteria as having mild non-proliferative diabetic retinopathy (NPDR), moderate or severe NPDR, or proliferative diabetic retinopathy (PDR). Macular thickness was measured using optical coherence tomography (OCT) (Cirrus High- Definition OCT; Carl Zeiss Meditec, Dublin, CA, USA) and axial length employing an IOL Master instrument (Carl Zeiss Meditec).

We classified DME patients as either IVB-responsive or -refractory. Responsiveness was defined as either CST < 300 μm or a CST reduction ≥ 50 μm at 1 month after 3 consecutive monthly injections of IVB.[18, 19]

### Assay of cytokines and growth factors

Concentrations of IL-1β, IL-2, IL-8, IL-10, IL-17, PlGF, and VEGF in 75 μL of aqueous humor from the anterior chamber (collected via anterior paracentesis during the first IVB injection or cataract surgery, and were immediately stored at -80°C until analysis) were measured. The assays used human antibodies against IL-1β, IL-2, IL-8, IL-10, IL-17, PlGF, and VEGF. The antibodies were immobilized on beads; 75-μL humor samples with 75 μL Calibrator Diluent RD6-52 were added to the bead preparations. The samples were incubated for 2 h at room temperature (20–25°C) after bead addition, for a further 1 h at room temperature after the addition of detection antibodies, and for 30 min at room temperature after the addition of streptavidin-phycoerythrin reagent. A Luminex-x-MAP suspension array system (Luminex, Austin, TX, USA) was used for detection; this is a multiplexed, microsphere suspension immunoassay that detects and quantitates spectrally unique microspheres attached to specific antibodies. The technique enables many samples to be analyzed in a single reaction.

The detection limits and dynamic ranges are as follows: 0.8 pg/mL with a dynamic range to 3,950 pg/mL for IL-1β, 1.8 pg/mL with a dynamic range to 8,510 pg/mL for IL-2, 1.8 pg/mL with a dynamic range to 1,140 pg/mL for IL-8, 1.6 pg/mL with a dynamic range to 890 pg/mL for IL-10, 1.8 pg/mL with a dynamic range to 2,090 pg/mL for IL-17, 1.9 pg/mL with a dynamic range to 470 pg/mL for PlGF, and 2.1 pg/mL with a dynamic range to 2,170 pg/mL for VEGF.

### Statistical evaluation

Statistical analyses were performed using SPSS for Windows software (ver. 20.0; SPSS, Chicago, IL, USA) and R (ver. 3.2.3, 2015-12-10, Platform: x86_64-redhat-linux-gnu, R Core Team (2015) [R: A language and environment for statistical computing. R Foundation for Statistical Computing, Vienna, Austria.URL https://www.R-project.org/.])

We used the t-test, Mann—Whitney U-test, and the chi-squared test to compare the values and the ratio of the participants groups. The Wilcoxon signed-rank test was used to compare changes in IOP, CST, and BCVA, after placement of intravitreal dexamethasone implants. Linear regression analysis was employed to identify CST-associated factors. Logistic regression was employed to identify factors associated with responsiveness to IVB injection. The statistical significance level was set at p<0.05.

## Results

The average age of the cataract patients was 67.92 ± 13.03 years and that of the DME patients was 56.81 ± 7.96 years (p = 0.004). There were 30 males and 34 females in the study group and 8 males and 5 females in the control group. There was no significant difference in either axial length or initial IOP between the study and control groups. Levels of IL-2, IL-8, PlGF, and VEGF were significantly elevated in the DME group (p = 0.007, p < 0.001, p < 0.001, and p = 0.004 respectively) (Table 1).

**Table 1:** Demographics and baseline clinical characteristics of all study participants.

	DME group (n = 64)	Control group (n = 13)	p-value
Age (years)	56.81 ± 7.96	67.92 ± 13.03	0.004
Sex (male: female)	30:34	8:5	0.377
IOP (mmHg)	14.61 ± 3.21	14.15 ± 3.21	0.642
Axial length (mm)	23.44 ± 0.81	23.20 ± 0.84	0.343
IL-1β level (pg/mL)	3.49 (1.86;3.49)	3.04 (1.86;3.49)	0.343
IL-2 level (pg/mL)	55.65 ± 21.54	36.91 ± 25.56	0.007
IL-8 level (pg/mL)	17.71 (12.81;28.66)	0.00 (0.00;3.94)	<0.001
IL-10 level (pg/mL)	0.00 (0.00;0.79)	0.00 (0.00;1.52)	0.861
IL-17 level (pg/mL)	2.56 (0.96;2.96)	1.59 (0.96;2.56)	0.192
PlGF level (pg/mL)	1.52 (0.82;2.52)	0.47 (0.47;0.65)	<0.001
VEGF level (pg/mL)	70.13 (37.33;105.45)	38.90 (25.66;55.54)	0.004

Values are expressed as mean ± SD or median and interquartile range, as appropriate

Abbreviations: DME, diabetic macular edema; IOP, intraocular pressure; IL, interleukin; PlGF, placental growth factor; VEGF, vascular endothelial growth factor

In the subgroup analysis, there was no significant difference in levels of aqueous cytokines and responsiveness of IVB between PDR and NPDR patients (Table 2).

**Table 2:** Demographics and baseline clinical characteristics of DME patients.

	NPDR group (n = 17)	PDR group (n = 47)	p-value
Age (years)	60.18 ± 7.90	55.60 ± 7.71	0.041
Sex (male: female)	7:10	23:24	0.777
IOP (mmHg)	14.18 ± 3.66	14.77 ± 3.06	0.521
Axial length (mm)	23.08 (22.83;23.72)	23.25 (22.98;24.30)	0.386
IL-1β level (pg/mL)	1.86 (1.86;3.49)	3.49 (2.90;3.49)	0.242
IL-2 level (pg/mL)	58.53 ± 20.57	36.91 ± 25.56	0.526
IL-8 level (pg/mL)	22.04 (15.05;33.66)	17.00 (12.27;25.11)	0.242
IL-10 level (pg/mL)	0.00 (0.00;1.15)	0.00 (0.00;0.34)	0.313
IL-17 level (pg/mL)	2.56 (0.96;3.37)	2.56 (0.96;2.56)	0.604
PlGF level (pg/mL)	1.34 (0.91;2.05)	1.52 (0.77;3.49)	0.498
VEGF level (pg/mL)	77.89 (58.24;84.27)	66.20 (35.88;113.98)	0.301
Preoperative BCVA (logMAR)	0.30 (0.20;0.50)	0.50 (0.20;0.70)	0.206
Postoperative BCVA (logMAR)	0.20 (0.20;0.30)	0.40 (0.20;0.60)	0.077
Preoperative CST (μm)	391.00 (363.00;401.00)	392.00 (352.50;471.00)	0.727
Postoperative CST (μm)	340.00 (263.00;392.00)	344.00 (294.00;418.50)	0.681

Values are expressed as mean ± SD or median and interquartile range, as appropriate

Abbreviations: DME, diabetic macular edema; IOP, intraocular pressure; IL, interleukin; PlGF, placental growth factor; VEGF, vascular endothelial growth factor, BCVA best-corrected visual acuity; CST, central subfield thickness

Preoperative CST was associated with preoperative BCVA and aqueous humor IL-10 level on multivariate regression analysis (p < 0.001, p = 0.006, respectively) (Table 3).

**Table 3:** Variables associated with the preoperative CST of DME patients as revealed by regression analyses.

	Univariate analysis		Multivariate analysis	
	ß ± SE	p value	ß ± SE	p value
Age (years)	0.087 ± 1.294	0.947		
Axial length (mm)	-15.667 ± 12.487	0.214		
DMR stage (mild NPDR to PDR)	0.750 ± 12.332	0.952		
DM duration (years)	-0.216 ± 1.305	0.869		
Preoperative BCVA (logMAR)	126.092 ± 34.433	< 0.001	116.185 ± 32.756	< 0.001
IL-1 ß level (pg/mL)	1.350 ± 6.779	0.843		
IL-2 level (pg/mL)	0.245 ± 0.477	0.610		
IL-8 level (pg/mL)	0.061 ± 0.490	0.901		
IL-10 level (pg/mL)	42.689 ± 14.229	0.004	37.791 ± 13.134	0.006
IL-17 level (pg/mL)	0.029 ± 5.461	0.996		
PlGF level (pg/mL)	-2.371 ± 3.024	0.436		
VEGF level (pg/mL)	-0.072 ± 0.160	0.654		

CST, central subfield thickness; DME, diabetic macular edema; IL, interleukin; NPDR, non-proliferative diabetic retinopathy; PDR, proliferative diabetic retinopathy; PlGF, placental growth factor; VEGF, vascular endothelial growth factor.

Of the 64 DME patients, 28 (43.75%) exhibited either CST < 300 μm or a reduction in CST ≥ 50 μm 1 month after the last IVB injection. On sub-group analysis, the mean IL-8 concentration of the refractory group was higher than that of the responsive group, and multivariate logistic regression analysis showed that the IL-8 was the only factor associated with responsiveness (OR = 0.95, p = 0.017) (Table 4).

**Table 4:** Results of logistic regression, effect on responsiveness to IVB.

	Responsive group (n = 28)	Refractory group (n = 36)	Univariate analysis		Multivariate analysis	
			Odds ratio (95% CI)	P value	Odds ratio (95% CI)	P value
Age (years)	56.50 ± 9.18	57.06 ± 7.00	0.99 (0.93, 1.06)	0.780		
Sex (male: female)	11:17	19:17	0.58 (0.21, 1.56)	0.285		
Axial length (mm)	23.50 ± 0.85	23.39 ± 0.80	1.17 (0.63, 2.19)	0.614		
BCVA (logMAR, baseline)	0.39 ± 0.20	0.50 ± 0.31	0.21 (0.03, 1.36)	0.111		
CST (baseline), (μm)	398.21 ± 63.79	429.89 ± 90.87	0.99 (0.99, 1.00)	0.127	0.99 (0.99, 1.00)	0.137
Diabetes duration (years)	11.25 ± 8.87	10.19 ± 7.14	1.02 (0.95, 1.08)	0.593		
IL-1β level (pg/mL)	3.46 ± 1.73	3.08 ± 1.33	1.18 (0.85, 1.68)	0.323		
IL-2 level (pg/mL)	56.22 ± 22.21	55.22 ± 21.32	1.00 (0.98, 1.03)	0.853		
IL-8 level (pg/mL)	16.81 ± 13.15	30.82 ± 23.96	0.95 (0.91, 0.99)	0.018	0.95 (0.91, 0.99)	0.017
IL-10 level (pg/mL)	0.39 ± 0.67	0.48 ± 0.69	0.81 (0.37, 1.70)	0.587		
IL-17 level (pg/mL)	2.26 ± 1.57	2.57 ± 2.11	0.91 (0.68, 1.19)	0.512		
PlGF level (pg/mL)	2.76 ± 3.38	2.87 ± 3.44	0.99 (0.85, 1.15)	0.890		
VEGF level (pg/mL)	83.90± 72.34	82.64 ± 58.29	1.00 (0.99, 1.01)	0.938		

**Fig 1:**
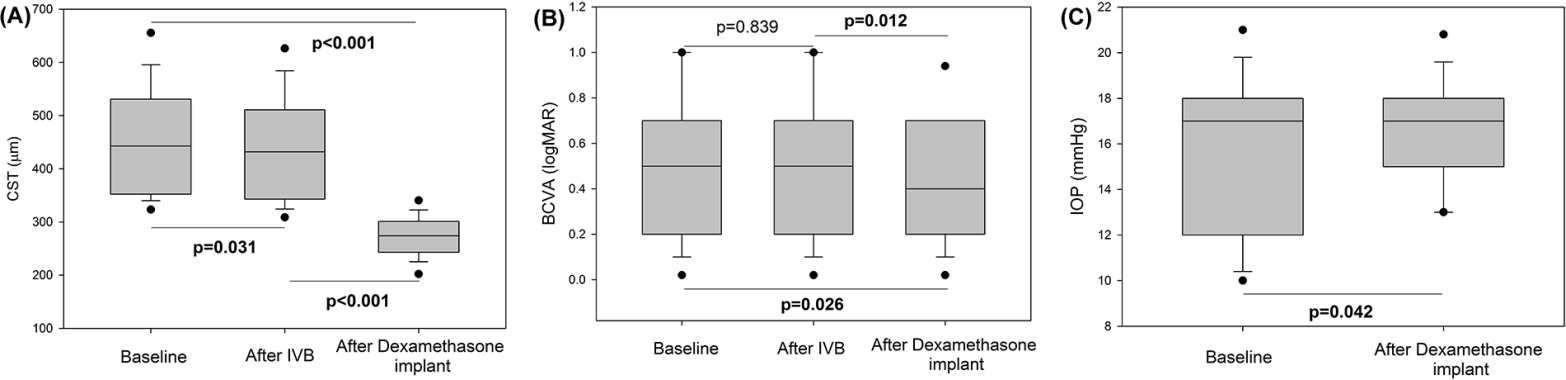
Box-and-whisker plots for central subfield thickness (CST), best-corrected visual acuity (BCVA), and intraocular pressure (IOP) changes in patients receiving dexamethasone implants due to poor responsiveness to 3 months of intravitreal bevacizumab (IVB) injections. The circles represent outliers. (A) CST was significantly reduced both after IVB injection and placement of dexamethasone implants. (B) BCVA did not improve significantly after IVB injection, but did upon dexamethasone treatment. (C) The baseline IOP was 15.52 ± 3.23 mmHg, and increased significantly to 16.61 ± 2.21 mmHg after placement of dexamethasone implants.

IVB, intravitreal bevacizumab; DME, diabetic macular edema; BCVA best-corrected visual acuity; CST, central subfield thickness; IL, interleukin; PlGF, placental growth factor; VEGF, vascular endothelial growth factor,

Of the 36 refractory patients, 23 received intravitreal dexamethasone implants. Of these, 17 (73.91%) exhibited CST < 300 μm and 20(86.96%) exhibited a CST reduction ≥ 50 μm at the 1-month follow-up. The CST and BCVA of 23 patients improved significantly compared with the values prior to implantation (Fig 1).

## Discussion

Many studies have found that DME is mediated by inflammatory cytokines and VEGFs, the actions of which are interconnected.[8, 20] Analysis of vitreous samples yields valuable information on retinal status,[21] but obtaining such samples is invasive and data quality may be compromised if the samples are contaminated with blood. Other studies have also shown that the aqueous humor reflects retinal status; levels of many cytokines are elevated during retinal hypoxia or inflammation.[17, 22, 23] Most studies on aqueous humor have simply shown that the concentrations of various materials differed between control and DME patients.[15, 24] Few studies have explored the associations between prognosis and individual cytokine levels.[25, 26] We thus sought associations between cytokine and VEGF levels, and responsiveness to anti-VEGF therapy. We first determined that IL-8 concentrations may be associated with anti-VEGF responsiveness.

IL-8 is a well-known pro-inflammatory cytokine that acts as a neutrophil chemoattractant and a T-cell activator.[27] In the eye, hypoxia induces IL-8 synthesis by endothelial and glial cells, associated with neovascularization in patients with ischemic retinal disease.[28, 29] Many studies have shown that elevated IL-8 levels in the aqueous humor of DME patients may be associated with inflammation-induced damage to the blood-retina barrier.[15, 30] One article has suggested that IL-8 may play a role in DME development that is not adequately controlled by anti-VEGF antibodies or steroids.[31] However, another study found that intravitreal triamcinolone was effective in patients unresponsive to IVB, and that the efficacy was associated with the IL-8 level in the aqueous humor.[32] This result matches our study, and we suggest that the DME more related with inflammation could manifest higher aqueous IL-8. This could be a good biomarker to predict responsiveness of therapeutic agents. The response rate of DME patients with IL-8 levels <17.71 pg/mL (the median value of DME patients in this study) was 19/31 (61.28%), but that of patients with IL-8 levels greater than the median value was only 7/31(22.81%). These proportions differed significantly (p = 0.004; odds ratio (OR) 5.429). The role played by IL-8 in DME requires further investigation.

IL-10 produced by activated macrophages and T-cells exerts various effects that are principally anti-inflammatory and immunosuppressive in nature.[33] In the eye, IL-10 may be associated with an angiogenetic response to hypoxia, but the details remain unclear.[34, 35] IL-10 levels in aqueous humor are elevated in patients with diabetes, retinal vascular occlusion, or primary intraocular lymphoma.[36–38] However, few IL-10 data are available for DME patients, and the results vary. One study reported that aqueous humor IL-10 levels correlated negatively with VEGF level,[36] and another study found that aqueous humor IL-10 levels were elevated in patients in whom DR progressed.[39] A recent study found that aqueous humor baseline IL-10 levels were negatively associated with BCVA.[17] Our data suggest that IL-10 may be associated with the CST of DME patients. Both BCVA and CST are markers of DME severity and disease progression. Like other studies, we found that CST was significantly associated with BCVA (p < 0.001, Table 3). Thus, IL-10 levels may also be associated with DME severity; more studies are required.

In the protocol H study of the Diabetic Retinopathy Clinical Research Network, mean reduction was 56 μm after two 1.25-mg IVB injections.[40] Although the OCT platforms used differed, we obtained a similar result after three consecutive IVB injections; the mean baseline and postoperative CSTs were 416.03 ± 81.14 and 362.23 ± 95.64 μm respectively; the mean reduction was thus 53.80 ± 63.35 μm. The protocol T study showed that, after 1-year IVB treatment, CST decreased by 101 ± 121 μm on average.[12] However we did not continue IVB until 1year and performed intravitreal dexamethasone implants in some of the refractory patients. The mean CST reduction after implantation, compared with the baseline level, averaged 178.22 ± 107.41 μm, and visual acuity improved significantly (Fig 1). The IOP increment was only 1.09 ± 2.41 mmHg; no patient exhibited IOP > 21 mmHg. However, in the first year of the BEVORDEX study, IOP elevations ≥ 5 mmHg from baseline were evident in 46% of patients, and a two-grade rise in cataract density was reported in 13% of patients receiving dexamethasone implants.[14] Thus, both the IOP and visual acuity require long-term follow-up.

Our study had certain limitations. First, the aqueous levels of only IL-2, IL-8, PlGF, and VEGF were significantly elevated in the DME group. This may be attributable to the small number of control patients, who differed in mean age from the DME group. Also, differences among the characteristics of patients enrolled in other studies may have affected our results. Second, the relationships between biomarker levels and fluorescein angiographic or OCT angiographic images should be studied in terms of DME pathogenesis. We plan a follow-up study along these lines. Third, a 1-month follow-up of patients receiving dexamethasone implants is too short to evaluate drug effects or side-effects. Changes in CST, BCVA, IOP, and cataract progression must be evaluated long-term.

In summary, the aqueous humor concentrations of IL-10 were associated with CST and those of IL-8 levels were associated with IVB responsiveness. Additional studies with more patients are required to confirm our results and to elucidate DME pathogenesis. Such studies may provide the basis for novel therapeutic approaches.
