# Correction: Aqueous humor cytokine levels in patients with diabetic macular edema refractory to anti-VEGF treatment

**DOI:** 10.1371/journal.pone.0207902

**Published:** 2018-11-16

**Authors:** Jin-woo Kwon, Donghyun Jee

The following information is missing from the Funding section: This study was funded by the National Evidence-based Healthcare Collaborating Agency (Grant Number: HC16C2299).
